# Multi-predator assemblages, dive type, bathymetry and sex influence foraging success and efficiency in African penguins

**DOI:** 10.7717/peerj.9380

**Published:** 2020-06-30

**Authors:** Grace Sutton, Lorien Pichegru, Jonathan A. Botha, Abbas Z. Kouzani, Scott Adams, Charles A. Bost, John P.Y. Arnould

**Affiliations:** 1 School of Life and Environmental Sciences, Faculty of Science & Technology, Deakin University, Burwood, Victoria, Australia; 2 Centre d’Études Biologiques de Chizé, UMR7372 CNRS/Univ La Rochelle, Villiers-en-Bois, France; 3 DST/NRF Centre of Excellence at the FitzPatrick Institute of African Ornithology, Institute for Coastal and Marine Research, Department of Zoology, Nelson Mandela University, Port Elizabeth, South Africa; 4 Marine Apex Predator Research Unit (MAPRU), Institute for Coastal and Marine Research, Department of Zoology, Nelson Mandela University, Port Elizabeth, South Africa; 5 School of Engineering, Deakin University, Geelong, Victoria, Australia

**Keywords:** Penguin, Prey capture, Benthic, Camera, Group foraging, Accelerometer, Bio-logging, Endangered, South Africa, Sex-specific foraging

## Abstract

Marine predators adapt their hunting techniques to locate and capture prey in response to their surrounding environment. However, little is known about how certain strategies influence foraging success and efficiency. Due to the miniaturisation of animal tracking technologies, a single individual can be equipped with multiple data loggers to obtain multi-scale tracking information. With the addition of animal-borne video data loggers, it is possible to provide context-specific information for movement data obtained over the video recording periods. Through a combination of video data loggers, accelerometers, GPS and depth recorders, this study investigated the influence of habitat, sex and the presence of other predators on the foraging success and efficiency of the endangered African penguin, *Spheniscus demersus*, from two colonies in Algoa Bay, South Africa. Due to limitations in the battery life of video data loggers, a machine learning model was developed to detect prey captures across full foraging trips. The model was validated using prey capture signals detected in concurrently recording accelerometers and animal-borne cameras and was then applied to detect prey captures throughout the full foraging trip of each individual. Using GPS and bathymetry information to inform the position of dives, individuals were observed to perform both pelagic and benthic diving behaviour. Females were generally more successful on pelagic dives than males, suggesting a trade-off between manoeuvrability and physiological diving capacity. By contrast, males were more successful in benthic dives, at least for Bird Island (BI) birds, possibly due to their larger size compared to females, allowing them to exploit habitat deeper and for longer durations. Both males at BI and both sexes at St Croix (SC) exhibited similar benthic success rates. This may be due to the comparatively shallower seafloor around SC, which could increase the likelihood of females capturing prey on benthic dives. Observation of camera data indicated individuals regularly foraged with a range of other predators including penguins and other seabirds, predatory fish (sharks and tuna) and whales. The presence of other seabirds increased individual foraging success, while predatory fish reduced it, indicating competitive exclusion by larger heterospecifics. This study highlights novel benthic foraging strategies in African penguins and suggests that individuals could buffer the effects of changes to prey availability in response to climate change. Furthermore, although group foraging was prevalent in the present study, its influence on foraging success depends largely on the type of heterospecifics present.

## Introduction

Within the marine environment, unpredictable or cyclical changes may cause fluctuations in the availability and distribution of resources (Fauchald, 2009; Hoskins et al., 2008). In response, marine predators show spatio-temporal flexibility in their at-sea movements and foraging decisions in order to increase the probability of resource acquisition (Hull, 2000; Weimerskirch, 2007). Marine predators may target aggregations of predictably abundant prey, arising from physical processes such as upwellings, fronts and eddies (Bost et al., 2009; Dragon et al., 2010). However, the location of these prey can change seasonally and be affected by oceanographic variation, which is thought to affect foraging success (Inchausti et al., 2003). Knowledge of how marine predators use cues in their environments to find food is fundamental to understanding their influence on prey populations, other predators’ populations and ecosystem functioning. Correspondingly, determining the factors that influence the foraging behaviour of marine predators is crucial to predicting their possible responses to environmental variability (Grémillet & Boulinier, 2009).

For air breathing marine predators, foraging strategies are also expected to reflect the vertical distribution of their prey. For instance, highly nutritious pelagic prey often occur in spatially and temporally unpredictable distributions, with prey captures by pelagic foragers occurring at a broad range of depths in response to this (Miramontes, Boyer & Bartumeus, 2012). In contrast, benthic foragers exhibit “square-shaped” dives which are determined by slope of the ocean floor, with consecutive dives being similar depths and duration (Elliott et al., 2008; Tremblay & Cherel, 2000). Benthic diving is often considered more energetically costly and individuals must maximise the time spent at the bottom of the dive in order to increase foraging success (Costa et al., 2004). However, the benthic diving is thought to be more profitable for some species due to a lack of pelagic prey or difficulties capturing pelagic prey (Arnould & Hindell, 2001). Furthermore, the trade-off between physiological diving ability and predictability of prey availability seems to be a motivating factor for the benthic foraging strategies present in some animals.

Group foraging is adopted by many pelagic foraging marine predators due to the patchy distribution of their prey (Pöysä, 1992; Silverman, Veit & Nevitt, 2004; Weimerskirch et al., 2010). In contrast, for benthic foragers, less information is known regarding group foraging behaviour. It is hypothesised that it is less likely due to benthic prey being solitary, more uniformly distributed or not requiring concentration (Casaux & Barrera-Oro, 2006). However, aggregations of prey sometime occur in benthic habitats which can cause the assemblage of marine predators to shallow benthic environments (Takahashi et al., 2008; Tremblay & Cherel, 2000). Previous studies have highlighted conflicting outcomes of group behaviour on foraging success. For example, where some species may be seen to benefit from group foraging due to information transfer or prey capture facilitation (Thiebault et al., 2014) others suggest that group foraging is just a response to the spatially aggregated prey, leading to intra-specific competition (Handley et al., 2018; Sutton, Hoskins & Arnould, 2015). While group foraging is thought to benefit some, less is known about how multi-predator feeding associations may influence prey capture success. As the predicted shortage of pelagic prey is thought to increase group size and foraging associations, understanding how the presence of conspecifics and heterospecifics may influence the foraging success of individuals is crucial to identifying shifts in behaviour in response to a changing environment.

The development and miniaturisation of behaviour data loggers has enabled insights into the distribution and foraging behaviour of air-breathing marine predators (McIntyre, 2014; Wakefield, Phillips & Matthiopoulos, 2009). Animal-borne video cameras used in conjunction with these behavioural data loggers can provide context-specific information for cryptic at-sea behaviours (Thiebault et al., 2014; Watanabe & Takahashi, 2013). In particular, they may reveal the complex relationships between individuals, conspecifics and heterospecifics during foraging and how changes in the abundance and distribution of prey may result in different behavioural responses (Sutton, Hoskins & Arnould, 2015; Takahashi et al., 2003). However, very little is known how group behaviour influences individual foraging effort, success and efficiency. Such information is crucial for predicting how changes in prey availability due to environmental variability may influence the foraging decisions of predators.

African penguins (*Spheniscus demersus*) are endemic to Africa and classified as ‘Endangered’ due to a recent rapid population decline (Crawford et al., 2011; IUCN, 2019). Typically thought to be pelagic foragers, their survival and breeding success has been considered intrinsically tied to the availability of pelagic shoaling fish species such as anchovy (*Engraulis encrasicolus*) and sardine (*Sardinops sagax*) within their foraging range (Crawford, 1998; McInnes et al., 2017b). During the breeding season, their foraging range is reduced to <30 km from the colony in due to the need to return regularly to feed their offspring (Pichegru et al., 2009). Consequently, the presence of pelagic fish in proximity to the colony is of high importance.

Individuals on the west and south-west coast of South Africa are influenced by the productive waters of the Benguela upwelling region (Pichegru et al., 2009). Group foraging in these animals has been shown to positively influence foraging success (McInnes et al., 2017a). In contrast, there is less information regarding the factors influencing African penguins breeding on the south-east coast of South Africa in waters of lower productivity (Van Eeden et al., 2016). Furthermore, the prevalence of group foraging behaviour, and its consequences, on the south-east coast African penguin population is not known. Group foraging is suspected to be common, although recent decreases in population size of colonies on the south-east of South Africa raises concerns about Allee effect on foraging success (Ryan, Edwards & Pichegru, 2012). Knowledge of the foraging strategies employed by individuals in a region of comparatively low productivity could provide insights into how the species may respond to changes in prey availability. This is especially important given that over half of the African penguin population resides on islands in this region, an area known for its high degree of environmental variability (Van Eeden et al., 2016).

The aims of the present study were to, through use of a video cameras and accelerometry, classify prey captures in African penguins and determine (1) the possible use of the benthic environment and (2) the factors influencing prey capture success, foraging effort and diving efficiency.

## Materials & Methods

### Study site and data collection

**Figure 1:**
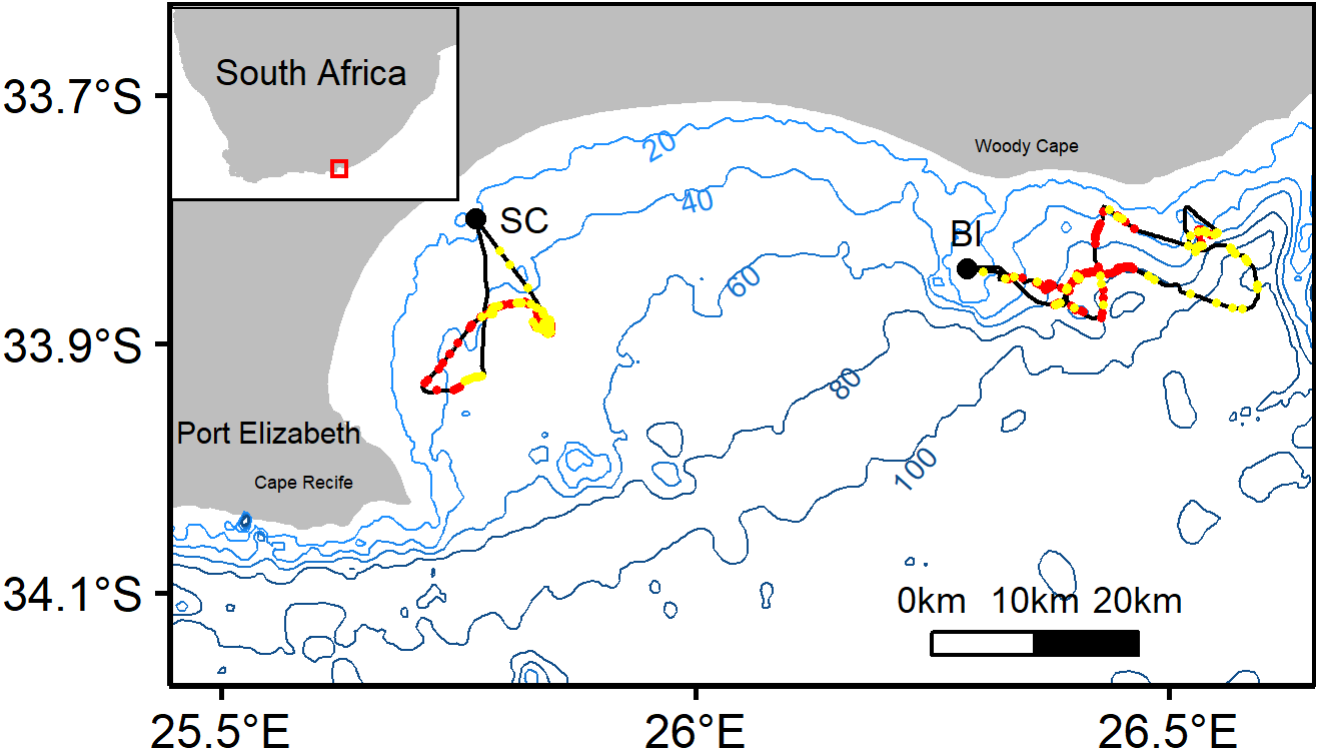
Representative tracks from African penguin study colonies St. Croix (SC) and Bird Island (BI). Red and yellow dots along the track indicate benthic and pelagic prey captures, respectively. Bathymetry provided in 20 m isobaths. Red box on inset map indicates the location of Algoa bay along the south-eastern coast of South Africa.

This study was approved by South African National Parks (SANParks) (PICL1282), the Department of Environmental Affairs (DEA, Res2017-76) and Nelson Mandela University (NMMU-A15-SCI-ZOO-008). Fieldwork was conducted during April-June 2017 at two African penguin colonies in Algoa Bay, St Croix (SC, 33°48′S, 25°46′E) and Bird Island (BI, 33°50′S, 26°17′E) (Fig. 1). These islands are the eastern-most breeding colonies for the species, hosting approximately half of the global population (Crawford et al., 2011). BI is the eastern-most colony of African penguins and supports approximately 2,500 individuals (DEA unpublished data, 2017). SC is a small (12 ha), rocky island and the world largest African Penguin colony, supporting 7,200 breeding pairs in 2017 (DEA unpubl. data).

Data collection occurred during the guard stage when breeding partners alternate between foraging trips to sea and guarding their chicks on land. Following a change-over, the partner departing to sea was captured at the nest and weighed using a digital scale (±10 g). Individuals were then instrumented with three devices for a single foraging trip: a GPS (Mobile Action Technology, I-gotU, GT-120, 44.5 × 28.5 × 13 mm, 20 g) which sampled at 1 min intervals and was packaged in heat shrink tubing for waterproofing; a combined tri-axial accelerometer and depth recorder (Technosmart, Axy-Depth, 12 × 31 × 11 mm, 7.5 g) which sampled acceleration and depth at 25 Hz and 1 Hz, respectively; and a video data logger (Catnip Technologies, Bird Cam, 25 × 45 × 15 mm, 30 frames per second, 24 g including housing), housed in a custom made 3-D printed plastic case, which recorded on a 30 min on, 30 min off schedule.

Devices were deployed before sunset with the cameras programmed with a start delay of 12 h to avoid recording on land, at night or during periods close to the colony. The devices were attached to feathers along the dorsal mid-line using waterproof tape (Tesa 4651, Beiersdorf, AG, GmbH, Hamburg) with the camera placed in front of the other devices so as to streamline the package and minimise logger effects on diving behaviour. Together, the package was <3% body mass (average body mass = 3,127 ± 63 g) and <0.3% cross-sectional surface area (average cross sectional surface area = 152 ± 20 mm). Efforts were made to minimise disturbance and reduce animal handling time with all procedures lasting <10 min. After deployment, the individual was released back to its nest where it remained until voluntarily departing for a foraging trip.

Individuals were recaptured after a single foraging trip and the devices were removed. Mass was recorded again and morphometric measurements were taken with Vernier callipers (bill length, bill depth, bill width; ± 0.1 mm) and a metal ruler (flipper length; ± 1 mm). Sex of each individual was determined by discriminant function analysis (Pichegru et al., 2013).

### Data processing and statistical analyses

Unless otherwise stated, all data processing and statistical analyses were performed in the R statistical environment, version 3.5.2 (R Core Team, 2018). The GPS tracks were filtered by removing points located on land and those that exceeded their mean maximum speed of 12.4 km h^−1^, (Wilson, 1985). Retained GPS locations accounted for >97% of the filtered foraging trip and the average location distance between consecutive points along a track was 43.4 ± 4.8 m (max 8940.5 m). To account for the irregularity of GPS locations, all tracks were interpolated to 1 min intervals using the package *adehabitatLT* (Calenge, 2011). Dive data obtained from depth recorders were corrected for depth drift using *diveMove* package (Luque & Fried, 2011) and the following dive parameters were calculated for each dive: beginning and end of a dive; dive duration; dive depth; duration of descent; bottom time; ascent duration; and post-dive interval. Total vertical distance travelled (maximum depth multiplied by two) and dive rate (number of dives divided by foraging trip duration) was estimated for each individual trip.

Data with a depth of <1.5 m were removed in order to remove surface or transiting periods. For each dive, depth profiles were categorised as either benthic or pelagic following a two-step criterion. Benthic dives were first identified by calculating the ratio between the recorded maximum dive depth divided by the bathymetric depth (bathymetry obtained from GEBCO 15-arc second dataset at a resolution of 0.05°) at the approximate location of the dive (Baylis et al., 2015). Secondly, the depth of each dive was compared to the depth of the previous and following dive. All dives within 10% of either the previous or following dive and with a bathymetric depth ratio of >0.8 were classified as a benthic dive while the remaining dives were classified as pelagic (Baylis et al., 2015). Benthic dives in shallow waters (i.e., <15 m) or close to the colony were removed from further analysis as the resolution of bathymetry is not accurate in these conditions. In addition, visual inspection in IgorPro using Ethographer (Wavemetrics Inc, Portland OR, USA, version 6.3.7.2) was conducted and benthic dives could be characterised as trapezoid shape with a steady descent, horizontal bottom phase and a steady ascent (Tremblay & Cherel, 2000).

In order to calculate foraging effort per dive, the tri-axial acceleration data were filtered using a 1 s running mean to separate static acceleration (due to the animal position in space in respect to gravity) from dynamic acceleration (accounting for animal movement). Vectorial Dynamic Body Acceleration (VeDBA), a proxy for whole body activity (Qasem et al., 2012), was calculated for each dive using the equation:

\begin{eqnarray*}& & \mathrm{V eDBA}=\sqrt{ \left( {X}_{dyn}^{2}+{Y}_{dyn}^{2}+{Z}_{dyn}^{2} \right) } \end{eqnarray*}

where *X*, *Y* and *Z* are the dynamic acceleration (*dyn*) of horizontal (surge), vertical (heave) and lateral (sway) movements, respectively. The total and mean VeDBA were calculated for each dive and used as an index of energy expenditure.

Video data were viewed frame-by-frame and coded using a custom video annotation sheet created in Solomon Coder (Budapest, Hungary, Version 16.06.26). Video camera data was used to determine prey type, prey capture events and whether they occurred in the presence of other foraging predators. Position in the water column (diving or resting on the surface) was also recorded and used to align the camera data to the accelerometer, depth and GPS data in IgorPro (Wavemetrics Inc, Portland OR, USA, version 6.3.7.2) using Ethographer (Sakamoto et al., 2009).

In order to evaluate prey captures over the full foraging trip, a two-class Support Vector Machine (SVM) (Meyer & Wien, 2015), a type of pattern classifier, was used to detect potential prey captures from swimming periods. This was developed using positive prey capture signatures detected in accelerometer data and confirmed by video camera footage. A total of 1,648 prey capture events were identified from the video data and a sample of data at a ratio of 5:1 swim:prey capture was taken to train the model. This was done in order to reduce computing time for R software but maintain variation so as to retain the predictive power of the dataset and enable the accurate detection of prey captures across all individuals (Lee & Mangasarian, 2001). As in previous studies (e.g., Carroll et al., 2014; Chessa et al., 2017), statistics were extracted using a rolling window of 8 data points (corresponding to approximately 0.32 Hz) and were used to train the model. Where previous studies used ODBA as a proxy for energy expenditure, the current study used VeDBA as it may be a better proxy due to the slightly lower placement of the accelerometer in relation to the bird’s centre of gravity (Qasem et al., 2012). The model was developed using a 10-fold cross validation where data were randomly split 70:30 (training:testing) and the range of parameters which showed the highest overall accuracy were retained (Table 1; see Text S1 for further information).

**Table 1:** Results of cross validated SVM model used to detect swimming behaviour vs. prey captures events in African penguins. Model utilized a radial kernel and was 89% accurate in correctly identifying between swim and prey capture events with a false 11% positive rate.

**Metric**	**Mean ± S.E. (%)**
Accuracy	89 ± 0.07
Recall	75 ± 0.07
False positive rate	11 ± 0.3

For the prey captures over the full foraging trip as determined using the SVM, a Generalised Linear Mixed Models (GLMM) using a binomial error distribution with a logistic link function were employed to determine the factors influencing the likelihood of a successful foraging dive. Initial inspection of data indicated probability of success could be both colony- and sex-linked. As such, a Bernoulli response variable represented dives resulting in prey capture(s) (1) or no prey capture (0). This was fitted against the predictor variables: dive type (benthic or pelagic), sex and a three-way interaction between colony, dive type and sex.

While tri-axial accelerometers have been used to measure energy expenditure during foraging in numerous species (Grémillet et al., 2018; Jeanniard-du Dot et al., 2017; Qasem et al., 2012), validation of such a relationship in African penguins has yet to be determined. Therefore, to investigate the factors influencing foraging effort, the following 2 metrics were used per dive: Foraging Efficiency Index (FEI, prey captures/total VeDBA); and Dive Efficiency (DE, prey capture/dive duration). Linear Mixed Effects Models (LME) were fitted using the *lme4* package (Bates et al., 2014). The FEI and DE were log transformed in both models in order to normalise positively skewed data. Predictor variables included in the model were dive type (benthic or pelagic), colony and dive depth.

Separate LMEs were also performed for foraging behaviour periods with concurrent video data in order to investigate the influence of other predators on the FEI and DE. FEI and DE were modelled against the three class factor presence of other predators (solitary, seabirds and multi-heterospecifics) with individual bird as a random factor.

For each model, model selection was undertaken using Akaike Information Criterion scores corrected for small sample sizes (AICc), calculated for all combinations of the predictor effects using the dredge function in the package *MuMIn* (Barton & Barton, 2020). A subset of the most parsimonious models was identified as those with a ΔAIC of less than or equal to 4 and used to generate model averaged coefficient estimates. Mann–Whitney U test was performed to compare foraging trip parameters between sexes or colonies and LMEs were used to compare diving metrics between colonies and between sexes. Individual bird ID was used as a random factor to account for repeated measures in all models presented.

Unless otherwise stated, results are expressed as Mean ± SE.

## Results

Video, accelerometer, diving and GPS data loggers were deployed on a total of 18 breeding African penguins, 9 from SC and 9 from BI. However, due to water damage, video data were obtained from 14 individuals and GPS, accelerometer and dive recorder data was obtained for 17 individuals (Table 2). The average foraging trip lasted 23.09 ± 1.83 h during which individuals completed 576.41 ± 2.13 dives. On average, individuals spent 7.73 ± 1.01 h diving with depths averaging 14.46 ± 0.19 m (maximum = 90.56 m), and travelled total horizontal distances of 53.8 ± 5.1 km at a speed of 2.33 ± 0.07 km h^−1^, covering total vertical distances of 17.28 ± 2.87 km. There was no significant difference in horizontal distances travelled (*W* = 23, *P* = 0.24), vertical distances travelled (*W* = 33, *P* = 0.81) or trip duration (*W* = 34, *P* = 0.88) between colonies in this study.

### Fine-scale diving behaviour and foraging success

**Figure 2:**
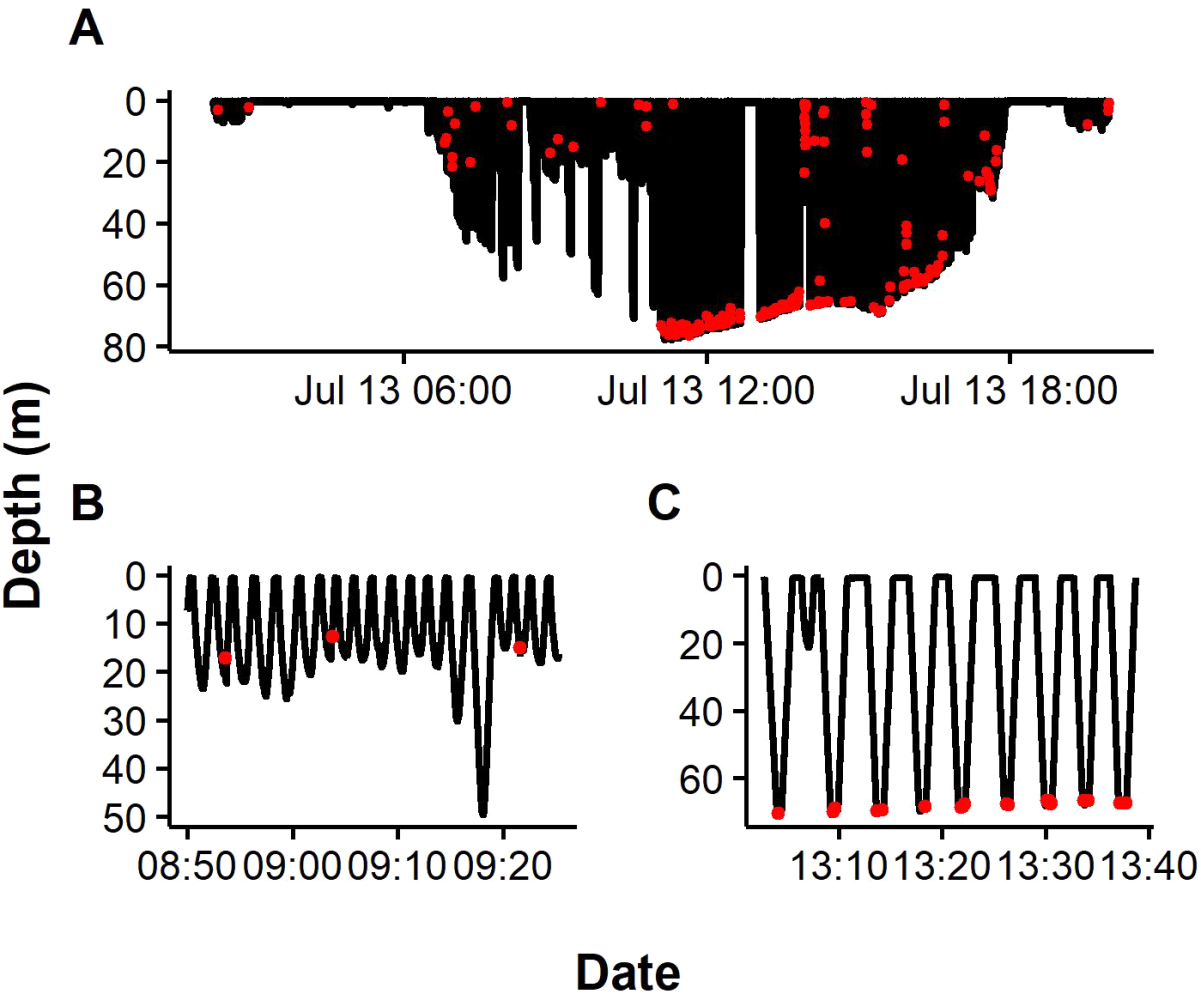
Representative dive profiles of African penguins from Algoa Bay. Red dots indicate prey capture events predicted by Support Vector Machine (SVM). Insets indicating pelagic (B) and benthic dives (C).

A total of 10029 dives were recorded, however by removing dives <1.5 m and those close to the colony (representing transiting periods), 6775 foraging dives remained, comprising of 3097 from BI and 3678 from SC (Table 2). Analysis of dive profiles in relation to bathymetry revealed penguins completed both pelagic and benthic dives. All individuals completed benthic and pelagic dives and the proportion of benthic dives for was slightly higher at SC with 31.4% of dives classified as benthic compared to 20.6% at BI (Table 2, SI3). Application of the SVM to the entire foraging trip identified capture signals throughout the water column on pelagic dives and during the bottom phase of benthic dives (Fig. 2). A total of 2,264 and 2000 prey captures were recorded during benthic dives and pelagic dives, respectively, equating to a mean of 10 ± 14.5 prey h^−1^. Interestingly, 60% of total benthic dives were successful whereas only 23% of all pelagic dives were successful (Table 2). Prey capture rate for benthic dives was 1.2 ± 0.1 prey dive^−1^ compared to 0.4 ± 0.1 prey dive^−1^ for pelagic dives.

**Figure 3:**
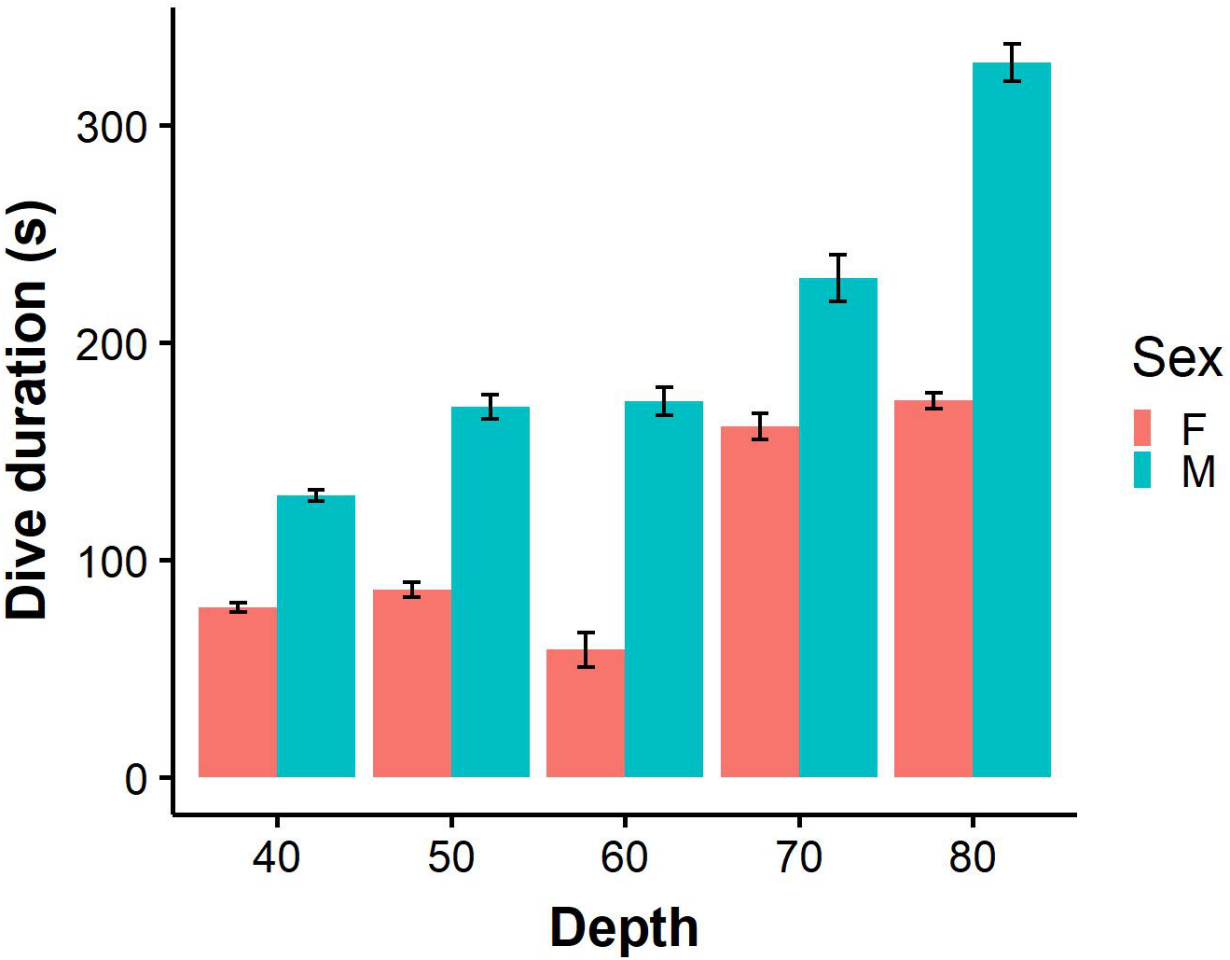
Dive durations of benthic dives (binned into 10 m intervals) performed by male (*N* = 9) and female (*N* = 7). Error bars represent standard error (S.E.) from the mean.

For benthic dives, individuals dived to a maximum depth of 91.5 m (mean 53.0 ± 0.9 m) at BI and 60.6 m (mean 40.3 ± 0.3 m) at SC. The most common mean maximum depth on benthic dives was 41.7 ± 1.7 m for females and 58.8 ± 1.0 m for males at BI, while females and males at SC dived to 36.8 ± 0.6 m and 43.22 ± 0.2 m, respectively. Males performed significantly longer benthic dives than females (Fig. 3; LME: *F* = 4.7, *P* < 0.05), and spent almost twice as long on the bottom phase (LME: *F* = 8.2, *P* < 0.5; Table 2). On benthic dives, there was a strong positive correlation of 0.91 between capture depth and maximum dive depth (Fig. S1) indicating that individuals captured on the bottom phase of the dive. Only 11.3% benthic dives were of incidences where individuals captured prey in the pelagic zone after performing a benthic dive.

**Table 2:** Dive and foraging efficiency indices for female and male African penguins rearing chicks on Bird Island (BI) and St Croix Island (SC). Parameters for benthic (B) and pelagic (P) dives are provided where applicable.

	BI				SC			
	Female		Male		Female		Male	
	*N* = 4		*N* = 4		*N* = 4		*N* = 5	
Adult mass (kg)	2.85 ± 0.06		3.26 ± 0.14		3.06 ± 0.13		3.30 ± 0.06	
Vertical travelled distance (km)	15.5 ± 4.0		22.5 ± 6.8		13.2 ± 4.1		16.9 ± 1.2	
Dive rate (dive/h^−1^)	22.7 ± 3.9		27.4 ± 5.1		25.2 ± 8.2		29.8 ± 6.4	
Dive type	B	P	B	P	B	P	B	P
Dives (*n*)	170	1,366	454	1,132	392	1,169	680	1,412
Total captured prey	159	655	595	381	482	466	992	428
Successful dives (%)	45.3	27.5	61.5	23.2	66.7	23.6	62.9	18.8
Mean capture depth (m)	46.0 ± 2.0	20.0 ± 0.6	57.5 ± 1.0	17.1 ± 1.0	28.4 ± 0.9	7.3 ± 0.4	38.0 ± 0.5	9.1 ± 0.5
Mean bottom time (s)	8.7 ± 1.2	10.3 ± 0.3	16.5 ± 1.0	14.8 ± 1.2	15.0 ± 0.9	8.4 ± 0.2	24.7 ± 1.6	7.9 ± 0.2
Prey capture rate (captures/dives)	1.02 ± 0.1	0.35 ± 0.1	1.41 ± 0.1	0.25 ± 0.1	1.25 ± 0.1	0.35 ± 0.1	1.61 ± 0.1	0.24 ± 0.1
Mean VeDBA	0.19 ± 0.1	0.20 ± 0.1	0.17 ± 0.1	0.24 ± 0.1	0.16 ± 0.1	0.24 ± 0.1	0.17 ± 0.1	0.27 ± 0.1
Total VeDBA	392.4 ± 10.0	158.3 ± 3.0	493.7 ± 8.1	138.8 ± 3.4	329.3 ± 4.2	101.6 ± 2.1	415.6 ± 3.1	87.0 ± 1.7

For pelagic dives, individuals at BI dived significantly deeper (16.6 ± 0.2 m; max: 79.7 m) than individuals at SC (8.2 ± 0.1 m, max: 40.2 m; LME: *F* = 9.0, *P* < 0.5). On successful dives prey capture rate was 1.6 ± 0.02. Irrespective of dive mode, males completed longer duration dives than females (male: 55.7 ± 0.9 s female: 37.7 ± 0.5 s). Contrary to previous studies (Pichegru et al., 2013), males had a slightly higher dive rate of 28.7 ± 4.0 dives h^−1^ compared to females, 23.9 ± 4.2 dives h^−1^. However, this difference was not significant (*P* > 0.05).

**Figure 4:**
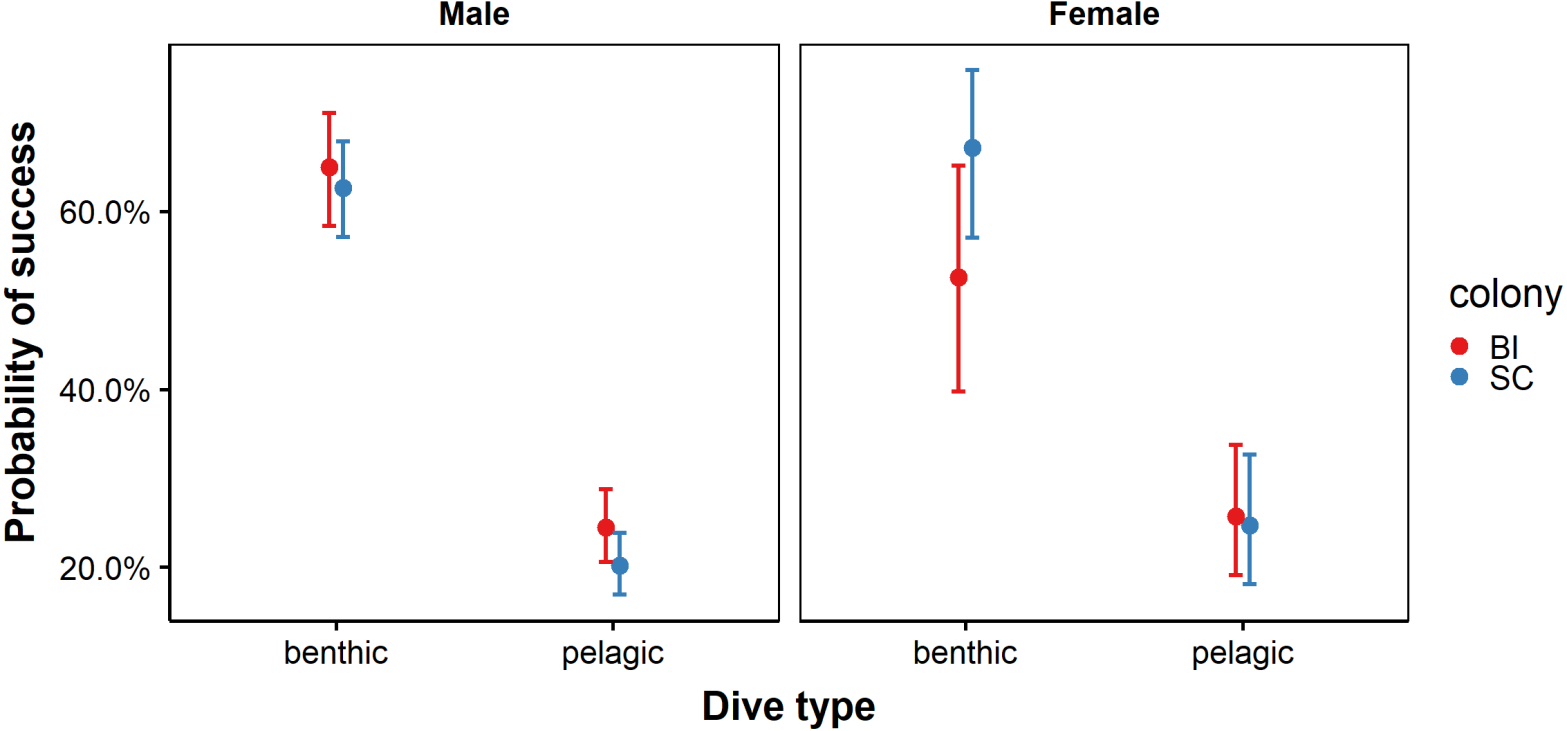
Probability of success for benthic and pelagic dives performed by male and female African penguins at Bird Island (BI) and St Croix (SC) colonies. Error bars represent standard error (S.E.) from the coefficient.

Candidate subsets explaining the probability of prey capture success comprised a total of ten models (Table S1). The most likely set of models retained the predictor effects sex, dive type, colony and an interaction terms between all predictors (Table 3). The probability of success was lower on pelagic dives compared to benthic dives. However, this relationship was stronger at SC than at BI. While sex-specific differences in foraging success were evident, they appeared to be dependent on colony and dive type. Males from both colonies performed equally well at benthic dives. Similarly, females from both BI and SC showed similar success rates for and pelagic dives. Males at BI were more likely to be successful on pelagic dives than males at SC, while females at SC were more likely to be successful at benthic dives than females at BI (Fig. 4). While model averaging retained sex, colony dive type and all possible interaction terms in the most parsimonious model, confidence intervals crossed zero for all parameters except dive type and an interaction between colony and dive type indicating that these two variables alone were good predictors of probability of success.

**Table 3:** Model summary tables for probability of prey capture success, Foraging Efficiency Index (FEI) and Dive Efficiency (DE) as predicted by Support Vector Machine (SVM) and as observed on camera. The abbreviations SC (St Croix), P (pelagic), F (female) and the symbol “:”, indicating an interaction between explanatory variables, are provided where possible. “Seabirds” and “multi” indicate where seabirds or seabird, fish and sharks were present during feeding events, respectively.

Response variable	Explanatory variables	Estimate	S.E.	*Z*∕*t* value	*P* value	CI (upper, lower)
Probability of Success	Intercept	0.45	0.15	2.93	**<0.01**	0.15, 0.74
	Colony (SC)	0.18	0.20	0.92	0.36	−0.2, 0.56
	Sex (F)	−0.14	0.23	0.61	0.54	−0.59, 0.31
	Type(P)	−1.56	0.14	11.14	**<0.001**	−1.83, −1.28
	Colony (SC):Sex (F)	0.39	0.28	1.38	0.17	−0.16, 0.94
	Colony (SC):Type (P)	−0.35	0.14	2.47	**<0.01**	−0.62, −0.07
	Sex (F):Type (P)	0.27	0.17	1.56	0.12	−0.07, 0.61
	Colony (SC):Sex (F):Type (P)	−0.37	0.20	1.88	0.06	−0.75, 0.02
FEI (SVM)	Intercept	−5.7	0.07	−78.7	**<0.001**	−5.9, −5.6
	Type (P)	0.48	0.03	18.17	**<0.001**	0.4, 0.5
	Colony (SC)	0.40	0.10	4.1	**<0.001**	0.2, 0.6
DE (SVM)	Intercept	−4.2	0.21	−20.0	**<0.001**	−4.6, −3.8
	Type (P)	0.77	0.03	23.4	**<0.001**	0.7, 0.8
	Colony (SC)	0.20	0.29	0.7	0.48	−0.4, 0.7
FEI (camera)	Intercept	−5.16	0.08	−62.91	**<0.001**	−5.33, −5.0
	Present predators (seabirds)	0.08	0.05	1.56	0.1	−0.02, 0.18
	Present predators (multi)	−0.08	0.05	−1.79	0.07	−0.17, 0.001
DE (camera)	Intercept	−3.31	0.22	−15.49	**<0.001**	−3.78, −2.91
	Present predators (seabirds)	0.17	0.06	2.68	**<0.01**	0.05, 0.30
	Present predators (multi)	−0.19	0.06	−3.21	**<0.01**	−0.30, −0.07

**Notes.**

Significant predictor effects (*P* < 0.05) are indicated by bold text.

Indices of energy expenditure, total and mean VeDBA, were significantly higher during prey capture dives (339.3 ± 3.6 and 0.20 ± 0.01, respectively) than non-prey capture dives (120.6 ± 1.4 and 0.16 ± 0.01, respectively; LME: *F* = 4777.4, *p* < 0.001, LME: *F* = 1095.2, *P* < 0.001, respectively).

The most parsimonious model for FEI (prey captured/total VeDBA) included both dive type (benthic or pelagic) and colony, with FEI being higher for SC birds and generally higher during pelagic dives than benthic dives regardless of the colonies (Table 3). For DE (diving efficiency; prey captures/dive duration) only dive type was retained in the most parsimonious model. While there were some differences between individual total VeDBA, sex did not appear to be a significant factor in predicting FEI. For both FEI and DE models, a single candidate model was retained and model averaging was not deemed necessary.

### Prey types and interactions with other predators

The video camera programming schedule provided sampling at regular intervals throughout the entire foraging trips. A total of 5511 dives were observed in the video data obtained from 14 African penguins, representing 56.2% of all dives recorded. Individuals captured clupeoid fish (anchovy, sardine and red-eye round herring *Etrumeus whiteheadi*) and pipe fish (*Syngnathus* spp.) on pelagic dives. Jellyfish (phylum Cnidaria) were also present, but unlike in other penguin species (Thiebot et al., 2017) there were no capture attempts made by African penguins. Due to low light levels at depths >30 m, it was not possible to determine prey type during benthic dives however the video cameras could pick up the sound of chase and capture, similar to when the animals were on the surface. This, in conjunction with the high precision SVM was evidence enough to suggest that prey captures occurred close to, or along the sea floor.

**Figure 5:**
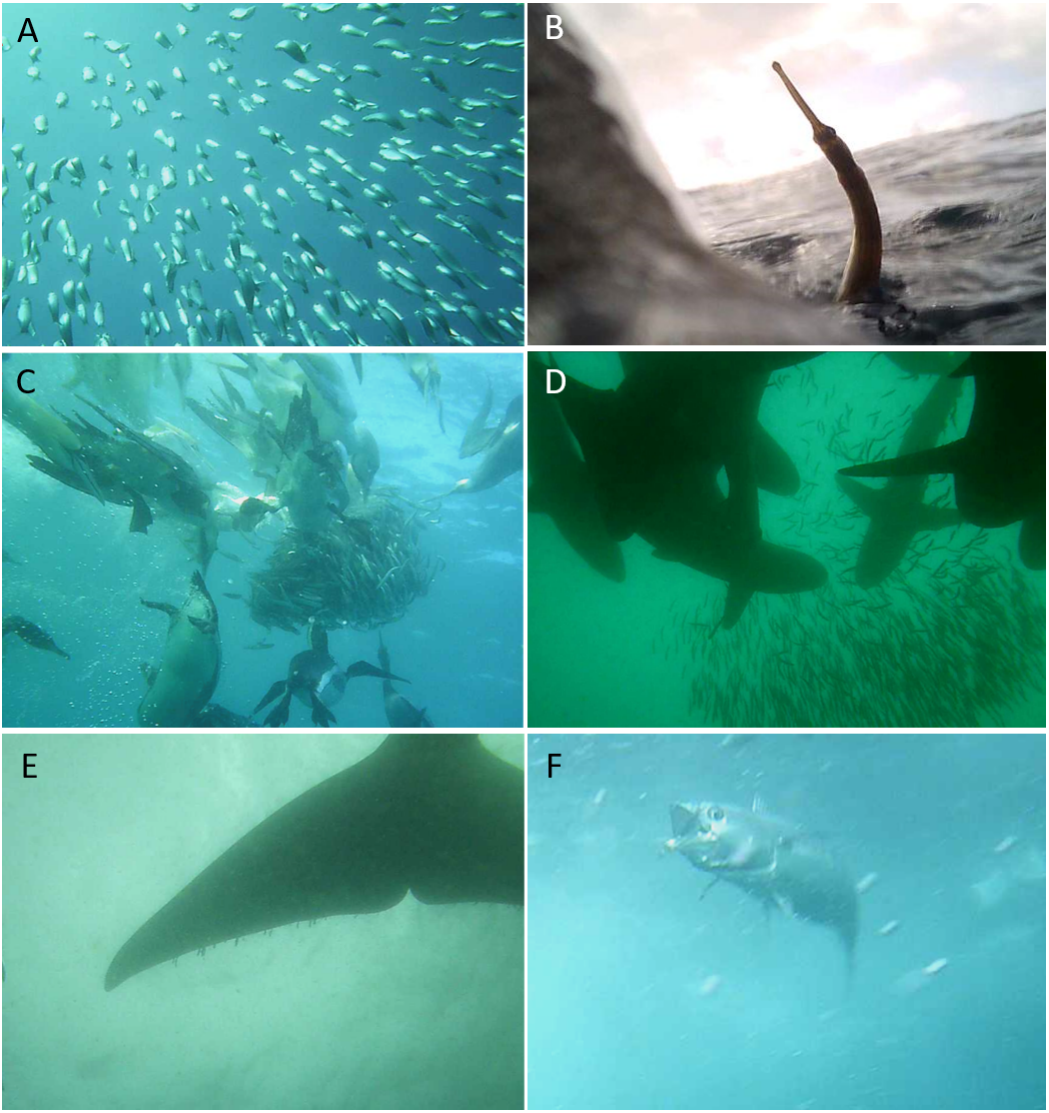
Representative still images of video data obtained from foraging African penguins. Bait fish (A); African penguin consuming a pipefish (B); Conspecifics and seabird heterospecifics (i.e., Cape Gannets *Morus capensis*) foraging on bait ball (C); Sharks (D); Whale (E) and Tuna (F).

Penguins were observed in multi-species feeding groups (in 30% of dives) with other seabirds such as Cape gannets (*Morus capensis*) and Cape cormorants (*Phalacrocorax capensis*), shearwaters (*Puffinus* spp.), terns (*Thalasseus* spp.) and conspecifics (Table 4; Fig. 5). Incidences of competition with other foraging seabirds (Cape gannet and Cape cormorant) were observed in which the penguin was unsuccessful in capturing prey in both events. Individuals also foraged in association with tuna (*Thunnus* spp.) and sharks (*Carcharhinus* spp.) on 11% of dives. The presence of sharks did not seem to influence the penguins’ behaviour, as individuals were observed moving between them and capturing fish escaping the bait ball (SI2). Bryde’s whales (*Balaenoptera brydei*) were observed on some video footage (*N* = 3 events) but, in these cases, the bait ball was quickly consumed and individuals were observed consuming only a single clupeoid fish on these occasions.

To investigate the influence of heterospecifics on foraging efficiency, foraging groups were separated into 3 levels: “none”, when individuals were foraging alone; “seabirds” when individuals were foraging in groups of other seabirds (including African penguins) and “multi-heterospecific” when individuals foraged in the presence of sharks and tuna, other seabirds were also present in these events. It was not possible to further separate the seabird group as penguins were rarely observed foraging in conspecific-only groups. Individuals in the presence of other predators exerted significantly more effort on successful prey capture events, than when alone (Table 4). Post-hoc tests indicated that there was a significant difference in mean VeDBA when individuals foraged alone, compared to foraging with other predators. However, there was no relationship between VeDBA and the type of predator present, with values similar for individuals foraging in seabird and multi-heterospecific groups (*P* > 0.05). Modelling indicated that neither the presence of seabirds nor multi-heterospecifics had a significant effect on FEI (Table 3). In contrast, DE was significantly affected both by the presence of seabirds and multi-heterospecifics. There was a significant increase in DE in the presence of seabirds and a decrease in the presence of multi-heterospecific groups.

**Table 4:** Total dives, number of prey captures, proportion of successful dives and average rate of activity (Mean VeDBA, *g*) for African penguins (*N* = 14) equipped with video cameras. Individuals foraged alone or in the presence of other seabirds or multi-heterospecific groups (i.e. combination of seabirds, sharks and tuna).

Predator presence	Total dives (% total dives)	Total prey caught	Proportion successful dives (%)	Mean VeDBA (*g*)
None	3,274 (59)	657	11.6	0.15 ± 0.01
Seabird	1,648 (30)	1373	44.2	0.20 ± 0.01
Multi-heterospecific	589 (11)	451	41.1	0.19 ± 0.01

## Discussion

Accurately determining where and when animals find and capture prey is integral to the study of foraging ecology. For diving marine predators, accurately detecting the location and depth of feeding events in the marine environment may provide information about the distribution of prey resources and the strategies by which animals obtain them (Viviant et al., 2010; Wakefield, Phillips & Matthiopoulos, 2009). In the present study, a unique combination of animal-borne video cameras, GPS, depth recorders and tri-axial accelerometers revealed new insights into the foraging behaviour and efficiency of an endangered marine predator, the African penguin. It was found that, while individuals performed both pelagic and benthic foraging dives, benthic diving was more successful in terms of the probability of prey captures, but successful pelagic dives were more energetically efficient. Modelling indicated success rates for pelagic dives were higher for females while males were more successful at capturing prey on benthic dives. However, differences in foraging success between colonies were observed, possibly due to the bathymetry surrounding each colony. Interestingly, camera footage revealed that African penguins’ diving efficiency increased with the presence of other seabirds but decreased in the presence of larger non-flying predators (sharks, whales, predatory fishes).

### Diving behaviour and foraging success

Linking diving behaviour and prey capture success is important for understanding the foraging behaviour of some marine predators. Accelerometry has been used in a range of studies to detect patterns of movement through unsupervised models because of the logistical difficulties of verifying datasets. In the current study, a supervised machine learning approach identified feeding events at sea from observations of African penguins wearing cameras and accelerometers. The model was developed from prey captures observed on pelagic dives as observing benthic prey captures was difficult due to low light conditions. However, studies that have used signals in accelerometry to detect prey captures suggest that prey capture signals remain the same, irrespective of depth or dive type (Volpov et al., 2015) but signals may change in duration or intensity with regard to prey type (Watanabe & Takahashi, 2013). The high accuracy of the SVM model for predicting potential prey captures highlights the beneficial use of accelerometers to calculate prey captures where cameras may be limited by battery capacity, or reduced light conditions during deep dives.

Diving behaviour varies within a species and may be dependent on habitat and prey availability, as well as sex or individual body size. In the present study, benthic foraging was more successful for males than females at BI only, which is likely due to a combination of differences in physiological diving capacities (as a result of sexual size dimorphism) and in exploited habitats. Indeed, male African penguins are generally larger than females which is thought to contribute to differences in foraging behaviour (Pichegru et al., 2013). Consequently, individuals with larger body sizes should have larger oxygen stores and, therefore, be able to dive for longer durations and reach greater depths (Lewis et al., 2005; Weise & Costa, 2007). The benthic environment surrounding BI is comprised of relatively steep sloping sea floor with depths exceeding 80 m, as such males may be more capable of exploiting these deeper benthic environments than females. In comparison, the seafloor surrounding SC Island is shallower, which may explain the similar success rates of females and males during benthic dives.

By contrast, females at both colonies showed similar probability of capture success during pelagic dives. Female success may be due to their overall smaller size, as smaller predators have shorter turning angles which may make them more manoeuvrable (Fish, Hurley & Costa, 2003) and may enable more efficient movement through the water while capturing highly mobile prey (Lovvorn et al., 2001). This may explain why males, who were highly successful benthic divers, also consumed pelagic prey when available, even though their capture success was lower than females.

Low prey capture rates on pelagic dives in this study may be due to the inclusion of shallower dives up to 1.5 m, which may be considered transiting dives. Previous studies in Algoa Bay have used a dive threshold of three metres in an attempt to remove transiting dives from analyses (McInnes & Pistorius, 2019). However, in the present study, 204 prey captures were observed within the first three metres of the water column which would have been omitted if a threshold of 3 m was used, accounting for just over 10% of the prey captures detected during pelagic dives. By setting the dive threshold too high, it is possible to miss prey captures, however setting the dive threshold lower, it may include travelling dives as foraging dives, thereby reducing pelagic dive success rate. Even though some prey captures were detected close to the surface of the water, the dive threshold of 1.5 m was selected in order to remove most transiting periods. However, increasing the dive threshold to 3 m only improved the proportion of successful pelagic dives from an average of 23.2 to 26.9%.

Benthic diving has been documented in several penguin species including Gentoo, *Pygoscelis papua* (Kokubun et al., 2010; Robinson & Hindell, 1996), Yellow-Eyed, *Megadyptes antipodes* (Mattern et al., 2007), Emperor, *Aptenodytes forsteri* (Rodary et al., 2000), Adelie, *Pygoscelis adeliae* (Ropert-Coudert et al., 2002), Chinstrap, *Pygoscelis antarcticus* (Takahashi et al., 2003) and macaroni penguins, *Eudyptes chrysolophus* (Tremblay & Cherel, 2000). However, no such diving behaviour has been previously reported for African penguins. Throughout their range African penguins appear to be entirely reliant on pelagic prey (Crawford et al., 2006; McInnes et al., 2017b; Petersen, Ryan & Gremillet, 2006), even in areas of low prey quality and abundance (Ludynia et al., 2010). Although prey capture events could be identified on benthic dives, it was not possible to identify the prey type being consumed due to low visibility. A single study, reported Beaked sandfish (*Gonorhynchus gonorhynchus*) and Barred fingerfin (*Cheilodactylus pixi*)*,* two benthic fish species, commonly presented in 9.6 and 4.2% of African penguin diet samples in individuals at SC and BI (Randall & Randall, 1986). Although it is probable that penguins are consuming some benthic species during benthic dives, pelagic prey have been known to occupy depths >70 m (Beckley & Van der Lingen, 1999; Roel & Armstrong, 1991), deeper than many parts of Algoa Bay. Hence, penguins foraging at these depths could be consuming pelagic species on benthic dives. Indeed, similar behaviour was observed in Cape cormorants foraging on the west and south western coast of South Africa who performed benthic dives, while diet samples indicated they foraged exclusively on pelagic prey items (Cook et al., 2012).

As females may be less successful on benthic dives, they may have a higher reliance on pelagic prey compared to males, thereby possibly being more sensitive to reduced pelagic prey availability. A male-bias in the adult sex ratio of African penguins exists due to a combination of male-biased chick production and female mortality (Spelt & Pichegru, 2017). The higher rates of female mortality (Pichegru et al., 2013) could be partially explained by their extra sensitivity to the recent decrease in small pelagic prey in South Africa (Pichegru et al., 2012). Similarly, in Magellanic penguins (*Spheniscus magellanicus*), mortality rate is higher for females when conditions are unfavourable, resulting in a male-skewed adult sex ratio and population decline in this species (Gownaris & Boersma, 2019). Certainly, this could be the case for African penguins, however due to low sample sizes in the present study, further assessments of sex-specific diving behaviour, particularly in years of low prey availability are needed to better understand how females may adapt to changing conditions.

### Foraging efficiency and implications

Sea-floor depth has been shown to influence prey availability and foraging efficiency in marine predators (Christophe et al., 2001; Yen, Sydeman & Hyrenbach, 2004). For instance, in little penguins (*Eudyptula minor*), individuals from colonies with shallower waters show lower diving effort (total diving duration per hour) compared to individuals from colonies with deeper waters (Meyer et al., 2017). In African penguins, indices of foraging effort in this study (mean and total VeDBA) were higher on benthic dives than pelagic dives, suggesting that individuals from colonies surrounded by steeper bathymetry may put in more effort to capture prey.

Individuals at the SC colony were more efficient in both pelagic and benthic strategies than individuals at BI possibly due to the shallower bathymetry associated with SC. For example, as the bathymetry was shallower, the energy expenditure from benthic dives was lower, hence overall energy efficiency was higher for individuals at SC. Furthermore, pelagic foraging efficiency was higher at SC and may suggest higher prey availability in the waters around this colony during the time of our study, or that individuals were more efficient at capturing prey. This is consistent with the prediction that colonies with shallow waters are more favourable to diving seabirds compared to deeper waters as prey is presumably less dispersed and, therefore, easier to catch (Meyer et al., 2017).

Environmental variability has led to divergent foraging behaviours in many marine predators (Arthur et al., 2016; Castillo-Guerrero et al., 2016; Waugh & Weimerskirch, 2003). The south and south-east region of South Africa is characterised by warmer and less productive waters compared to the west and south-west coast which are enriched by the productive Benguela upwelling (Kirkman et al., 2016).

African penguins breeding in Algoa bay experience highly variable changes in ocean temperatures which is thought to influence the availability of prey (Goschen et al., 2012; Schumann, Cohen & Jury, 1995). Collectively the islands and surrounding waters of Algoa bay also support a large biomass of marine predators, including half of the global African penguin population, two thirds of the global Cape gannet populations, several tern, cormorant and gull colonies (Crawford et al., 2009), as well as a variety of other resident (Kirkman et al., 2007) and migratory marine mammals (Melly et al., 2018; Reisinger & Karczmarski, 2010) foraging in the highly variable waters within the bay. As such, large amounts of inter- and intra-specific competition could also explain the occurrence of benthic diving within individuals from this site.

The fact that African penguins display benthic diving in the current study, but that no such diving behaviour is evident in individuals from the West coast, could indicate the ability to adapt foraging behaviour to buffer against the effects of foraging in an area of reduced pelagic prey availability. In periods of low productivity, individuals with a higher degree of flexibility in their foraging strategies are predicted to have a greater chance of surviving (Montevecchi et al., 2009; Quillfeldt et al., 2011). Although pelagic prey surveys (L Pichegru, unpublished data, 2017) and trip durations during the study year were similar to previous years, benthic diving may indicate that a degree of plasticity present in the foraging behaviour of African penguins. This may be useful in buffering the negative effects in years of low pelagic prey availability. However, more information is needed over multiple seasons to address this.

### Prey type and at-sea predator interactions

Recent deployments of miniaturised animal-borne video cameras on African penguins revealed that conspecifics group foraging behaviour increase individual foraging success by aggregating and pushing prey towards the surface (McInnes et al., 2017a). Other seabirds such as shearwaters and Cape cormorants have been shown to also benefit from African penguins driving aggregated prey towards the surface within their reach (McInnes & Pistorius, 2019). It was not known, however, whether African penguins themselves also benefit from the presence of such heterospecifics during pelagic foraging.

Competitive exclusion may occur when larger individuals/species outcompete and exclude smaller individuals/species for the same resources (Wilson, 1975). In the present study, African penguins foraged in the presence of a range of marine predators, including at least four species of seabirds, tuna, sharks and whales. While mixed feeding flocks have been observed during at-sea surveys (Ryan, Edwards & Pichegru, 2012), evidence of African penguins foraging in the presence of sharks has not been previously documented. In the present study, diving efficiency was lower when in the presence of sharks. This suggests that although sharks did not appear to deter African penguins from attempting to capture prey, the presence of larger predators may act as a physical barrier to the bait ball, forcing penguins to target prey escaping the school and preventing penguins corralling prey closer to the surface (Video S1). As such, individuals diving efficiency was lower as they could not gain access to the bait ball. Furthermore, lower efficiency observed in the presence of large apex predators may also reflect predator avoidance. Indeed, African penguins have been observed coming ashore with bites from larger predators (Johnson et al., 2006). Both competitive exclusion and predation avoidance could be composite factors in reduced foraging efficiency for African penguins when feeding in the presence of larger top order predators.

## Conclusions

In summary, African penguins breeding in Algoa Bay, South Africa, were observed to display sex–specific diving behaviours and foraging efficiency related to the bathymetry around their colonies. Individuals exploiting shallower sea-floor habitats may have a greater chance of capturing benthic prey. In addition, benthic foraging behaviour might buffer the effects of changes in pelagic prey availability, however this may come at a greater cost due to higher energy costs of benthic dives, especially for females which smaller size reduces their resilience. This study confirms the positive role conspecifics and other seabirds may have on African penguin individual foraging behaviour and efficiency. However, we revealed that the presence of larger predators decreased prey capture rates of African penguins, likely due to a combination of competitive exclusion and/or predation risk.

##  Supplemental Information

10.7717/peerj.9380/supp-1 Figure S1 Maximum dive depth against depth of capture of African penguins performing benthic dives (*r*^2^ = 0.91)

10.7717/peerj.9380/supp-2 Data S1 Accelerometry, dive data and modelling (including GPS locations) for African penguins

10.7717/peerj.9380/supp-3 Table S1 Model selection table for models determining factors influencing probability of success for African penguins performing benthic and pelagic dives Colony (St. Croix or Bird Island), Type (benthic or pelagic) and Sex (male or female) represented as “C”, “T” and “S”, respectively. “:” indicating interaction term between parameters.

10.7717/peerj.9380/supp-4 Text S1 Additional methods in developing Support vector machine (SVM) for detecting prey captures in African penguins

## Additional Information and Declarations

### Field Study Permissions

The following information was supplied relating to field study approvals (i.e., approving body and any reference numbers): Field experiments were approved by the South African National Parks (PICL1282).

### Data Availability

The following information was supplied regarding data availability: All data for African penguins contributing to the modeling results are available as Supplemental File. The supplementary video file is available at Figshare: Sutton, Grace (2020): African penguin Video S1.zip. figshare. Media. https://doi.org/10.6084/m9.figshare.12247055.v2.
