# Supplementary figures and images for: Multi-predator assemblages, dive type, bathymetry and sex influence foraging success and efficiency in African penguins
Source: PeerJ. 2020 Jun 30;8:e9380. doi: 10.7717/peerj.9380 (PMC7333648; doi:10.7717/peerj.9380)

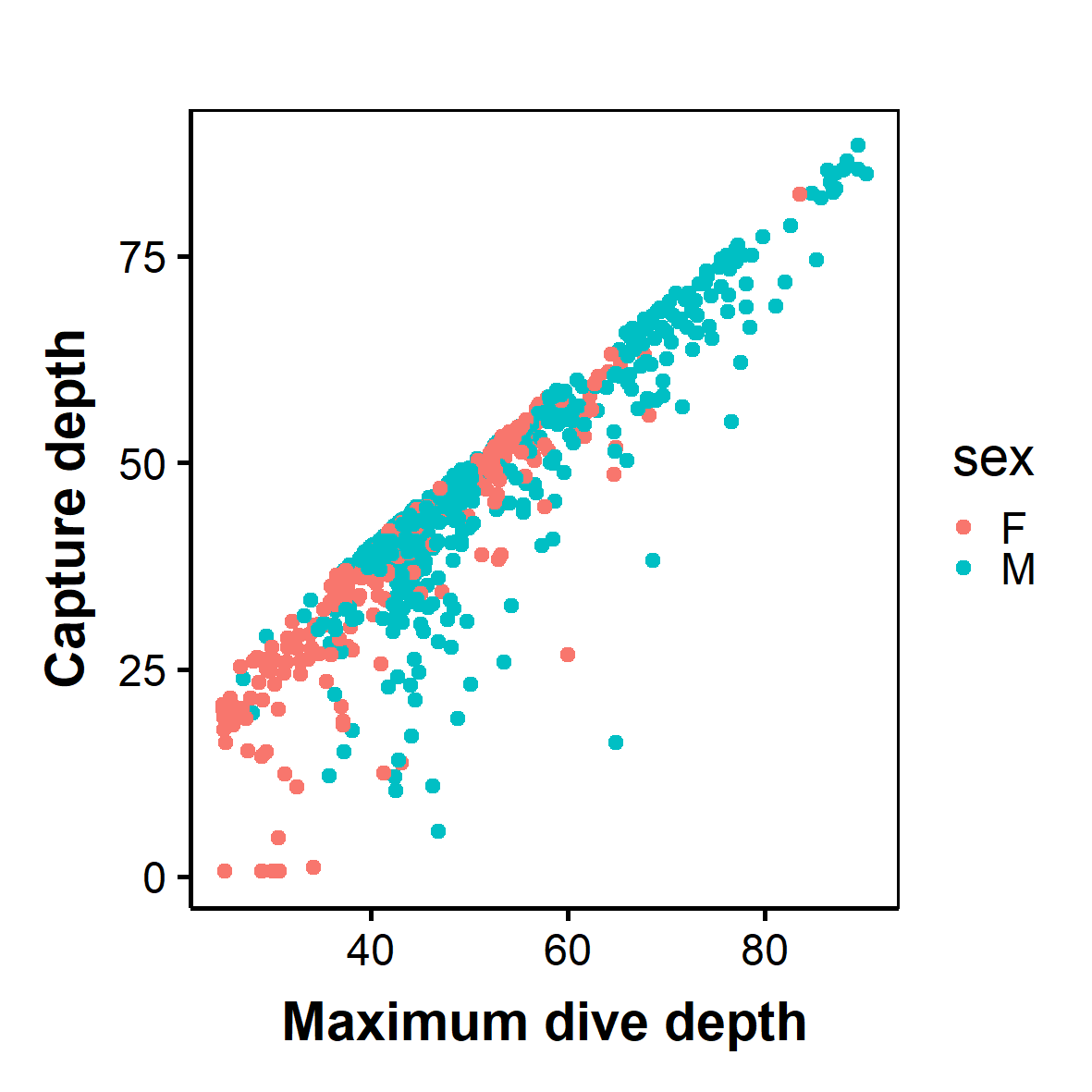

Supplement: Figure S1 [file peerj-08-9380-s001.png]
