# Supplementary material for: Multi-predator assemblages, dive type, bathymetry and sex influence foraging success and efficiency in African penguins
Source: PeerJ. 2020 Jun 30;8:e9380. doi: 10.7717/peerj.9380 (PMC7333648; doi:10.7717/peerj.9380)
Supplement: Table S1 — Colony (St. Croix or Bird Island), Type (benthic or pelagic) and Sex (male or female) represented as “C”, “T” and “S”, respectively. “:” indicating interaction term between parameters. [file peerj-08-9380-s003.docx]

**Table S1**. Model selection table for models determining factors influencing probability of success for African penguins performing benthic and pelagic dives. Colony (St. Croix or Bird Island), Type (benthic or pelagic) and Sex (male or female) represented as “C”, “T” and “S”, respectively. “:” indicating interaction term between parameters.

| Retained variables | df | logLik | AICc | ΔAIC | weight |
| --- | --- | --- | --- | --- | --- |
| C, S, T, C:S, C:T, S:T, C:S:T | 9 | -3813.2 | 7644.5 | 0.00 | 0.22 |
| C,T,C:T | 5 | -3817.4 | 7644.8 | 0.29 | 0.19 |
| C, S, T, C:T, S:T | 7 | -3815.6 | 7645.1 | 0.63 | 0.16 |
| C, S, T, C:S, C:T, S:T | 8 | -3815.0 | 7646.1 | 1.55 | 0.10 |
| C, S, T, C:T | 6 | -3817.2 | 7646.4 | 1.90 | 0.08 |
| T | 3 | -3820.2 | 7646.5 | 1.98 | 0.08 |
| S, T, S:T | 5 | -3818.7 | 7647.5 | 2.95 | 0.05 |
| C, S, T, C:S, C:T | 7 | -3816.8 | 7647.6 | 3.09 | 0.05 |
| S, T | 4 | -3820.0 | 7648.1 | 3.56 | 0.04 |
| C, T | 4 | -3820.2 | 7648.3 | 3.79 | 0.03 |
