# Supplementary material for: Multi-predator assemblages, dive type, bathymetry and sex influence foraging success and efficiency in African penguins
Source: PeerJ. 2020 Jun 30;8:e9380. doi: 10.7717/peerj.9380 (PMC7333648; doi:10.7717/peerj.9380)
Supplement: Text S1 [file peerj-08-9380-s004.docx]

**Text S1: Additional methods in developing Support vector machine (SVM) for detecting prey captures in African penguins.**

An SVM seeks to find the best “margin”, or kernel, that separates all positive (i.e. prey captures) and negative (i.e. swimming) events (Meyer & Wien 2015). After testing the suitability of a range of kernels (radial, linear, polynomial) the radial kernel was chosen as this model showed the highest overall accuracy. The model was further tuned by selecting the best values for gamma (how far the influence of a single training example reaches) and cost (controlling the trade-off between overfitting and under fitting of the support vector) and the following evaluation metrics were determined for each model: Accuracy - the number of correct predictions divided by the total number of predictions; Recall - the number of positive predictions divided by the number of total positive class values in the test data; and False positive rate -the number of incorrect positive predictions divided by the total number of positive predictions. The model with the highest accuracy and recall and the lowest false positive rate utilised a radial kernel where tuning parameters gamma and cost were 0.01 and 1, respectively (Table 1). This model was then applied to the whole foraging trip of each individual in order to identify potential prey capture events in the periods without corresponding video data. To avoid the misclassification, a positive signal (irrespective of it being detected in a benthic or pelagic environment) had to be detected in 3 or more consecutive rolling windows (>0.9 s).

Meyer D, and Wien FT. 2015. Support vector machines. *The Interface to libsvm in package e1071*:28.
